# Application of Vagus Nerve Branch Preservation in Thoracoscopic Surgery for Early-Stage Lung Cancer

**DOI:** 10.1155/2022/5143383

**Published:** 2022-04-11

**Authors:** Xiangzheng Liu, Zhimao Chen, Shijie Zhang

**Affiliations:** Department of Thoracic Surgery, Peking University First Hospital, Beijing 100034, China

## Abstract

**Background:**

In this study, we introduced a novel surgical strategy to protect vagal nerve branches during radical thoracoscopic surgery in right lung cancer and explored the effects of vagal nerve branch preservation.

**Methods:**

We retrospectively studied 53 patients with right-sided lung cancer with clinically staged T1N0M0 between 2019 and 2020. All 53 patients were treated with total thoracoscopic lobectomy and mediastinal lymph node dissection in the same number of lymph node stations. Of these, 22 patients adopted a vagus nerve branch protection strategy during lymph node dissection. Another 31 patients were treated with traditional lymph node dissection as the control group.

**Results:**

The characteristics of the patients were similar between the two groups. The operation time and intraoperative bleeding in the protection group were longer than those in the control group. However, the protection group had a lower average postoperative pain score and average postoperative hospital stay. The above difference was not statistically significant. Three cases of arrhythmia occurred in the protection group, including 1 case of tachycardia and 2 cases of atrial fibrillation. In the control group, 13 cases of arrhythmia occurred after the operation, including 8 cases of tachycardia and 5 cases of atrial fibrillation. We also tracked changes in the patients' heart rates throughout the treatment process (excluding patients with arrhythmias). An increased heart rate was observed postoperatively in both groups, but the increase of heart rate of the protection group was smaller than that of the control group; however, the difference was not statistically significant.

**Conclusions:**

A vagus nerve branch preservation-based approach to radical surgery is a safe and feasible strategy for right lung cancer treatment, which could significantly reduce the risk of postoperative arrhythmia in patients and may also have a potential role in reducing the length of hospital stay and maintaining heart rate stability in the postoperative period.

## 1. Introduction

Lung cancer has the highest morbidity and mortality among cancers worldwide. In 2012, 1.8 million people were newly diagnosed with lung cancer, and 1.6 million died from lung cancer [1]. Anatomic lobectomy and complete lymph node dissection have long been considered the standard surgical strategy for resectable non-small-cell lung cancer (NSCLC). Numerous retrospective studies have shown that limited resection, including wedge resection or segmentectomy, in stage IA disease may be equivalent to lobectomy, although their results were inconsistent. These bold strategies have introduced surgical possibilities for patients with more advanced ages or poor cardiopulmonary reserve [2]. With the advancement of minimally invasive surgery, many surgeons have endeavored to improve postoperative quality of life with a more minimally invasive and physiological technique. Over the past decades, the functions of the autonomic nervous system (especially for vagus nerves) have long been underappreciated in thoracic surgical procedures. The vagus nerve is the tenth cranial nerve, with mixed afferent (sensory), efferent (motor) nerve and parasympathetic nerve fibers, which innervate the principal organs, including the liver, lung, spleen, kidneys, and gut [3, 4]. The vagus nerve modulates the following: airway tone, perfusion, and secretion; breathing pattern; gastrointestinal secretion; and motility. It also plays an important role in regulating heart rate [3–5]. Vagus branches are often transected during traditional thoracic surgery involving mediastinal lymph node dissection, which is closely associated with postoperative cardiopulmonary and digestive system complications. Thankfully, vagus nerve functionality has gained attention in recent years, and vagus nerve branch sparing strategies have been explored during thoracic surgery procedures [6, 7]. However, most vagus nerve branch sparing strategies have been studied during thoracoscopic esophagectomy. Few researchers have focused on preserving the vagus nerve branches during radical surgery for lung cancer. Given the distinct differences in anatomical structure and surgical procedures between the left and right vagus nerve branches in the pleural cavity, in this study, we attempted to introduce a novel strategy of vagus nerve branch preservation in lymph node dissection for right lung cancer.

## 2. Methods and Materials

### 2.1. Clinical Characteristics

This is a retrospective study. We selected 22 patients treated by our group from 2019 to 2020, using vagus nerve branch preservation lymph node dissection as the experimental group. Considering that this is an exploratory surgical study. To avoid concerns about the possible effects of this procedure on lymph node dissection, the 22 patients we selected were all early stages of imaging staging. We selected 31 patients with the same stage at the same time and who used traditional surgical methods as the control group. The same treatment was adopted after the operation, a chest drainage tube was indwelled for postoperative observation, and the same criteria for removing the drainage tube were applied. We used a numerical rating scale to measure the patient's pain level, where 0 represents no pain and 10 represents the most severe pain.

### 2.2. Key Points of Surgical Skill

First, the right superior mediastinal pleura was incised to find the trunk of the vagus nerve. Sharp separation was used to dissect caudally following the vagus nerve trunk. Pulmonary branches were usually observed to arise from the trunk of the vagus nerve between the subclavian artery and azygos arch. They coursed along the trachea and then removed 2-3 branches to form the pulmonary plexus distally. Continuing to separate ventrally along the superior vena cava to the superficial area right of the aortic arch, the cardiac branches of the vagus nerve could be easily identified and should be preserved carefully. If the cardiac branches were given off inferiorly, separation following the vagus nerve trunk would probably be helpful to differentiate the origin of cardiac branches. After exposing and protecting the cardiopulmonary branches of the vagus nerve, the soft tissues, including lymph nodes, were dissected caudally until the upper edge of the azygos arch. Whether RUL lobectomy is involved or not, for complete resection of Station 4R, it is necessary to dissect from the right hilum to the cephalic side along the upper edge of the first branch of the right pulmonary artery. Then, they were separated through the upper and lower edges of the azygos arch, which could thoroughly expose the superior mediastinum and ensure complete resection of the lymph nodes. After exposure of the superior mediastinum, the descending track of the pulmonary branches was clearly visible and was carefully preserved.

**Figure 1:**
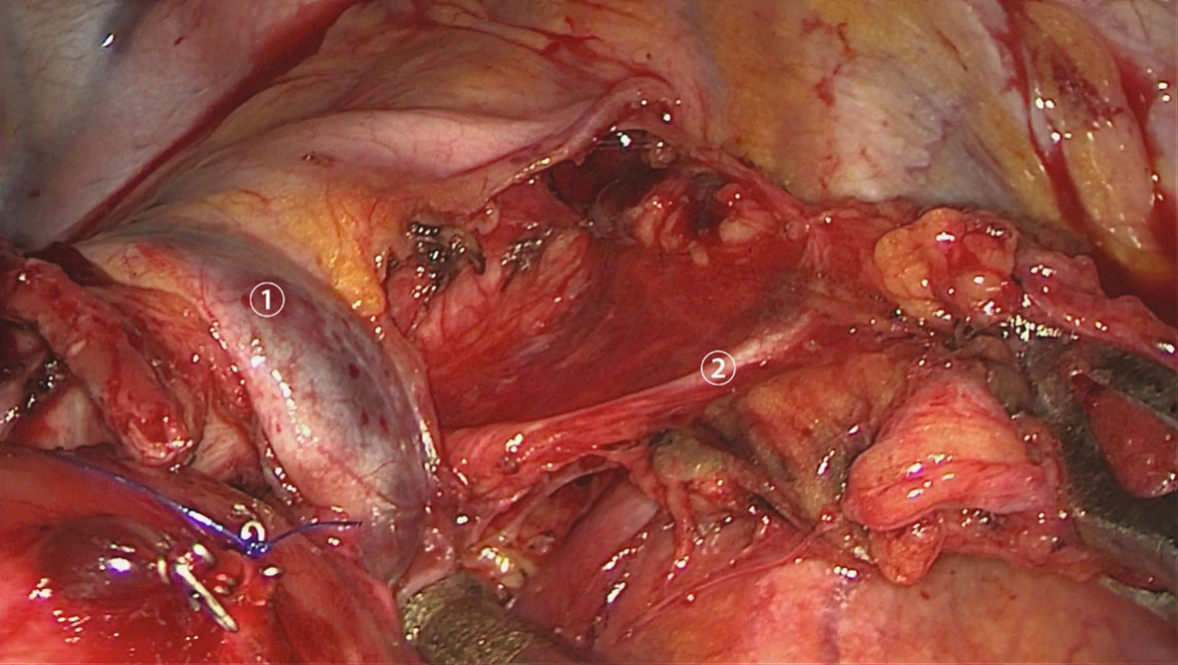
The pulmonary branch of the vagus nerve exposed during mediastinal lymph node dissection. ① Azygos vein. ② Pulmonary branch.

**Figure 2:**
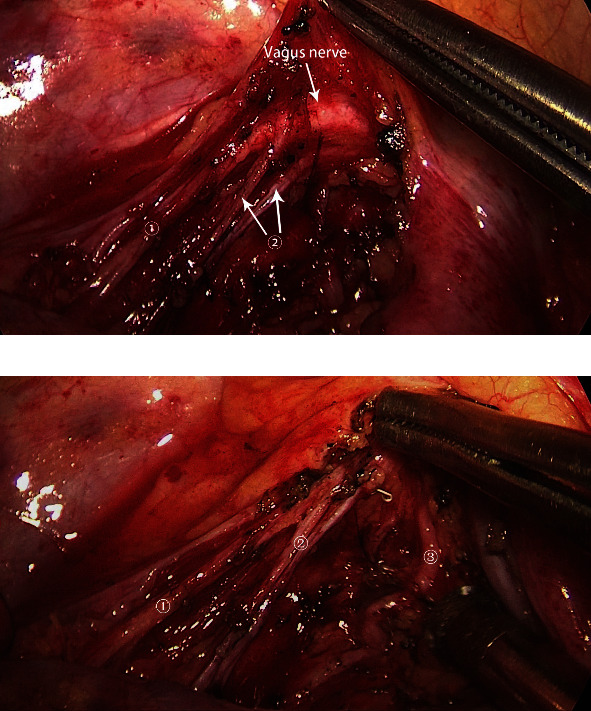
The main branches of the vagus nerve after mediastinal lymph node dissection. ① Vagus nerve. ② Pulmonary branch. ③ Cardiac branch. (a) and (b) were from the same patient.

**Figure 3:**
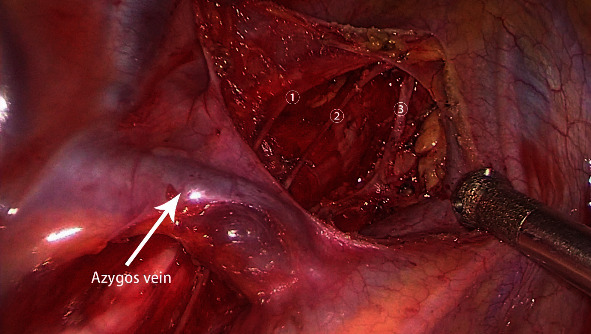
The main branches of the vagus nerve after mediastinal lymph node dissection.➀ Pulmonary branch. ② Pulmonary branch. ③ Cardiac branch.

Critical skills for vagus nerve branch preservation: the origin of pulmonary branches was obvious but thin in some cases. The pulmonary branches descended a distance to the main right bronchus and pulmonary hilum to form the pulmonary plexus. All these factors caused difficulty in preserving pulmonary branches. With sharp separation via scissors instead of energetic instruments, pulmonary branches were preserved in most cases. There is no need to worry about bleeding caused by sharp separation because there are just fine small vessels along the side of the area of pulmonary branches. In most conditions, bleeding in this area could be arrested via gauze compression. Generally, the cardiac branches were thick and located deeply, making them easier to preserve. However, if pulled roughly and energetic instruments, especially ultrasonic scalpels, are used, the risk of injury may increase.  Figure 1 shows the pulmonary branch of the vagus nerve exposed during mediastinal lymph node dissection. Figures 2 and 3 show the main branches of the vagus nerve after mediastinal lymph node dissection.

### 2.3. Follow-up

Heart rates were measured at 6 periods of time during the perioperative period: basal heart rate at admission, heart rate out of the postanesthesia care unit (PACU) after surgery, Day 1 after surgery, Day 2 after surgery, 2 days prior to discharge, and 1 day prior to discharge. Heart rate was measured 4 times in each period, and the average heart rates were calculated and recorded. Patients were required to sit in a calm state for half an hour before heart rate measurements via palpation or electrocardiogram (ECG). Drugs that might alter heart rate, such as calcium antagonists, beta-blockers, and bronchodilators, were ruled out during the perioperative period. When specific standards were met (i.e., no leak during coughing, light hemorrhagic drainage fluid, no fever, and drainage not exceeding 200 ml/day), the drainage tube was removed.

### 2.4. Statistical Analysis

Statistical descriptions included the mean ± SD or median (IQR) for continuous variables and frequency (n%) for categorical variables. The Mann-Whitney *U* test, t test, chi-square test, or Fisher's exact test were used to evaluate differences between groups with respect to their baseline characteristics. Two-way repeated-measures ANOVA was applied with change from heart rate as the dependent variable and groups, time, and groups multiplied by time interaction as independent variables. Statistical analysis was conducted using SPSS version 20.0 (IBM Co.; New York, USA). A *p* value of <0.05 was considered statistically significant.

## 3. Results

**Figure 4:**
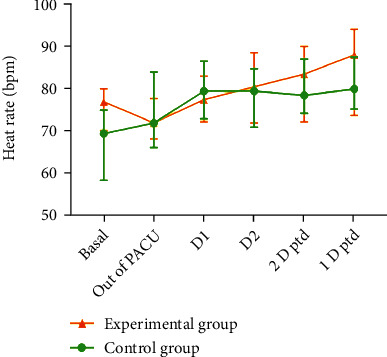
(Median IQR) Heart rate changes between the experimental and control groups. Definition of abbreviations: bpm: beats per minute; PACU: postanesthesia care unit; D 1: Day 1 after surgery; D 2: Day 2 after surgery; 2 D ptd: 2 days prior to discharge; 1 D ptd: 1 day prior to discharge.

**Figure 5:**
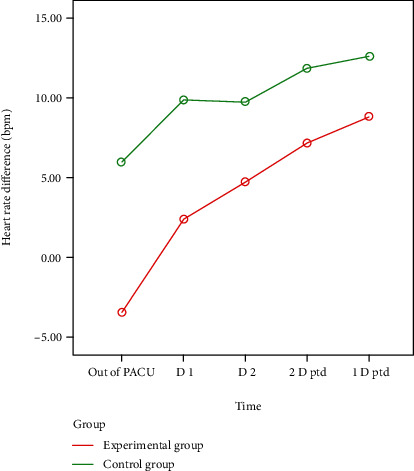
Estimated marginal mean heart rate difference compared to basal heart rate between the experimental and control groups. Definition of abbreviations: bpm: beats per minute; PACU: postanesthesia care unit; D 1: Day 1 after surgery; D 2: Day 2 after surgery; 2 D ptd: 2 days prior to discharge; 1 D ptd: 1 day prior to discharge.

There were no statistically significant differences in sex, age, smoking status, or comorbidities between the two groups. Detailed patient characteristics are shown in Table 1. Both groups of patients underwent thoracoscopic surgery and systematic lymph node dissection. The 22 patients in the protection group successfully retained the main cardiopulmonary branches of the vagus nerve. All patients were confirmed to have no pN staging. The average operation duration and blood loss of the protection group and the control group were 201.6 ± 49.6 min vs. 186.7 ± 40.2 min and 59.0 ± 25.7 ml vs. 58.0 ± 37.0 ml, respectively, and the difference was not statistically significant. The vagus nerve protection group had a slight advantage in regard to the postoperative pain score and the average hospital stay, but the difference was not statistically significant. In terms of complications, both groups of patients developed a certain amount of arrhythmia postoperatively, and 3 cases of arrhythmia occurred in the protection group, including 1 case of tachycardia and 2 cases of atrial fibrillation. In the control group, 13 cases of arrhythmia occurred after the operation, including 8 cases of tachycardia and 5 cases of atrial fibrillation. The difference in arrhythmia was statistically significant. For this reason, we also observed postoperative heart rate changes in patients without arrhythmia. The heart rate data of the two groups are expressed as the median (interquartile range, IQR), as shown in Table 2. The basic median of the protection group was higher than that of the control group, *p* > 0.05.  Figure 4 shows the change in heart rate (median (IQR)) between the protection group and the control group, which shows that in addition to the decrease in the heart rate of the protection group PACU at other points in time, the heart rate has an overall upward trend. The other perioperative periods were higher than the basal heart rate.  Figure 5 shows the estimated marginal average of the heart rate difference between the protection and control groups compared to the basal heart rates. The increase in the heart rate of the protection group was smaller than that of the control group, but the difference was not statistically significant.

## 4. Discussion

The vagus nerves leave the skull through the jugular foramen. They proceed caudally through the neck confined within the carotid sheath behind the internal carotid artery and internal jugular vein. The right vagus nerve gives off the right recurrent laryngeal nerve, which bypasses and is inferior to the subclavian artery [6, 8]. The main branches of the vagus nerve course caudally and medially through the superior mediastinum over the trachea and divide into several cardiopulmonary branches between the subclavian artery and azygos arch. Cardiac branches of the vagus nerve could arise from those superior cardiopulmonary branches coursing anteriorly to the trachea and posteriorly to the superior vena cava and be located deeply. If the branches leading to the heart were given off inferiorly, carefully following the main vagus nerve trunk and identifying branches going to the heart would probably be helpful to differentiate cardiac branches. The anterior pulmonary plexus forms just superiorly to the right pulmonary artery. Inferior to the azygos arch, the main branches of the vagus nerve pass posteromedially to the main right bronchus and pulmonary hilum, where they branch off, forming the posterior pulmonary plexus, which contains 77% (62–100%) of the total nerve supply of the right lung [6, 8, 9]. There are communicative branches between the anterior and posterior pulmonary plexuses. Some sympathetic and vagus nerve fibers can enter the lung through the pulmonary ligament. The right vagus nerve often branches to the front of the tracheal bifurcation and to the front of the left main bronchus. Consequently, incomplete anesthesia can result in a reflexive collapse of the left lung during right lung surgery through the above neural connection [10]. In clinical practice, the vagus nerve branches are not successfully preserved due to the considerable variation of the vagus nerve between patients and nonproficient operation skills, which is more common in the pulmonary branches. The cardiac branches are thick and located deeply. Generally, simple lymph node dissection procedures are less likely to cause cardiac branch injury. However, suppose the surgeon pulls roughly and uses electrosurgical instruments, especially ultrasonic scalpels. It may increase the risk of injury even though it can significantly shorten the operation time and simplify the operation steps. Common lung cancer postoperative complications related to intraoperative nerve injury include arrhythmia, chronic cough, hoarseness, pulmonary infection, and atelectasis. However, the probability of nerve injury during traditional pulmonary thoracoscopic procedures is low. Watanabe et al. [11] reviewed 992 patients with primary lung cancer who underwent thoracoscopic surgery for major pulmonary resection with mediastinal lymph node dissection (MLND). Fifteen of 992 patients (1.5%) experienced recurrent laryngeal nerve injury-related postoperative complications, and 3 (0.3%) experienced a bilateral vagal injury. However, this study lacked comparative assessments of the potential consequences of nerve damage through postoperative follow-up. In contrast, the rate of nerve injury is much higher during thoracoscopic esophagectomy. Teus J. Weijs et al. [7] revealed that nearly all vagus nerves to the right lung and inferior left lung lobe are transected during transthoracic esophagectomy.

Recent studies have shown that it is feasible to preserve a significant portion of the cardiopulmonary branches of the vagus nerve in lung cancer surgery. The main pitfall is leaving behind lymph nodes that might have been resected otherwise. Teus J. Weijs et al. [7] performed thoracoscopic esophagectomy while sparing the pulmonary vagus nerve branches in 10 human cadavers. In 8 of 10 cases, lymph nodes at Stations 10R and 10L were left behind, while lymph nodes at Station 7 were always removed. However, it is unclear whether residual lymph nodes are also present during traditional surgical procedures. Leaving lymph nodes behind when the vagus nerve branches are preserved will result in residual disease [7, 12]. Extensive CT screening significantly increased the detection of early-stage lung cancer [13]. Previous studies [14, 15] indicated that primary tumor size was a significant predictive factor for regional LN metastasis in early-stage lung cancer, and early-stage lung cancers with small tumor sizes tended to have a lower rate of lymph node metastasis. A retrospective study [15] including 354 patients with cT1N0M0 peripheral NSCLC showed that cT1a NSCLC had no hilar or intrapulmonary lymph node (LN) metastasis (0%), and NSCLC greater than 1 cm but less than or equal to 1.5 cm had significantly low rates of hilar or intrapulmonary (2.5%) and peripheral (2.5%) LN metastasis. Catching more early-stage cases with lower lymph node metastasis rates means a longer survival time for patients, which puts a higher demand for a more minimally invasive surgical technique to achieve a better postoperative quality of life. The clear and magnified visual field of thoracoscopy provides possibilities to achieve these goals. For early-stage lung cancers with small tumor sizes, it is feasible to preserve the main branches of the vagus nerve during radical surgical procedures. However, if lymph node metastasis is suspected preoperatively and/or enlarged or fused lymph nodes are observed intraoperatively, nerve preservation should not take priority over radical treatment.

The present study showed that compared with traditional surgery, the vagus nerve branch preservation-based lymph node dissection strategy required a longer operation time (201.6 ± 49.6 min vs. 186.7 ± 40.2 min) and resulted in slightly the same blood loss (59.0 ± 25.7 ml vs. 58.0 ± 37.0 ml); however, the difference was not statistically significant (see Table 1). These results suggest that a preservation-based vagus nerve branch radical surgery is a safe and feasible technique for right lung cancer treatment. Table 1 shows that compared with the control group, the postoperative pain score and hospitalization time (2.8 vs. 3.1; 5.2 vs. 5.8) of the protection group were lower, suggesting that the preservation of the vagus nerve branch may have a potential effect on reducing pleural effusion. In addition, it does not add to the pain of the patient's wound. We speculate that there are two possibilities. One is that the preservation of the vagus nerve branch itself will reduce the patient's pleural effusion, and the other is the restriction on the application of energy devices when operating around the vagus nerve, which reduces the stimulation of nearby lymphatic vessels and avoids thermal damage. Thereby reducing chest leakage, these two effects shorten the time required to use the drainage tube after the operation so that the patient can meet the discharge conditions earlier.

The most significant result of this study was a significant reduction in the number of postoperative arrhythmias (3 vs. 13). Postoperative arrhythmia is one of the most common complications after lung cancer surgery. It has always been highly related to preoperative complications, surgical methods, and surgical resection range. In the absence of significant differences in all known high-risk factors, we adopted this protective strategy to reduce the incidence of postoperative arrhythmia in patients. Arrhythmias are as long as few patients do not have obvious symptoms. Most patients will have symptoms such as panic and shortness of breath, which will significantly impact the patient's postoperative rehabilitation activities. Even a very small number of cases of malignant arrhythmias can induce serious complications such as thrombosis and shock. This affects the overall treatment effect of the patient. We speculate that this phenomenon is due to the integrity of the most likely preserved vagus nerve anatomy, thereby reducing the negative impact of surgery on nerve function. To further explore the possible mechanism, we also observed a slight increase in overall heart rate in both groups compared with preoperative basal heart rate (Table 2.). For the protection group, the heart rate decreased at the time-point out of the PACU in the protection group, and this result may be related to the effect of the volume state of the body, pain, and stress after surgery. In addition, due to the small sample size, individual outliers will also lead to large fluctuations in the overall data.  Figure 5 shows that under the background of the increased postoperative heart rate in both groups, the increment of heart rate of the protection group was smaller than that of the control group (*p* > 0.05), suggesting that the short-term effect of preserving the cardiopulmonary branches of the vagus nerve may be beneficial to the heart rate stability in the postoperative period and may have a potential preventive effect on postoperative arrhythmia.

This study's data suggests further prioritization of vagus nerve preservation in radical lung cancer surgery. After a review of related literature on anatomy, and based on our clinical practice, we confirmed the anatomical locations of vagus nerves that may be affected by lymph node dissection in right lung cancer surgery. For the right upper mediastinum, lymph node dissection in this area is usually an indiscriminate pursuit of “thoroughness”, which is not worth advocating today with the in-depth development of minimally invasive surgery and the popularization of the concept of enhanced recovery after surgery (ERAS). Many surgeons believe that this area does not require careful dissection because there are no important structures in this area, or that too much time should not be spent on lymph node dissection, which may lead to many unexplainable postoperative complications or discomfort in patients, and can even cause residual lesions. The autonomic nerve, including the vagus nerve and sympathetic nerve, plays a crucial role in regulating cardiopulmonary physiological functions [3, 4]. We tried to protect the autonomic nerve to reduce the influence on postoperative cardiopulmonary functions, including cardiac rhythm, chronic cough, blood pressure fluctuation, and pulmonary infection. At present, only preliminary results are available in this clinical research of our center, and the long-term outcomes still need to be further followed up.

## Figures and Tables

**Table 1:** Patient characteristics.

Variables	Experimental (*n* =22)	Control (*n* =31)	*p* value
Age (years, mean ± SD)	56.5 ± 8.8	58.3 ± 9.8	0.311
Gender			0.515
Male	10 (45.6%)	13 (41.9%)	
Female	12 (54.4%)	18 (58.1%)	
Smoking status			0.487
Smoker	4 (18.2%)	7 (22.6%)	
Nonsmoker	18 (81.8%)	24 (77.4%)	
Complications			
Coronary heart disease	2 (9.1%)	3 (9.7%)	0.662
Arrhythmia	3 (13.6%)	6 (19.4%)	0.437
COPD	4 (18.2%)	3 (9.7%)	0.309
Hypertension	8 (36.4%)	13 (41.9%)	0.123
FEV1 ≤ 2.0 L	4 (18.2%)	5 (16.1%)	0.563
Pa O_2_ ≤ 80.0	2 (9.1%)	2 (6.5%)	0.555
Tumor location			
RUL	14	17	
RML	2	4	
RLL	6	10	
Pathologic types			
Adenocarcinoma	21	28	
Squamous carcinoma	1	3	
Duration of operation(min, mean ± SD)	201.6 ± 49.6	186.7 ± 40.2	0.146
Blood loss(ml, mean ± SD)	59.0 ± 25.7	58.0 ± 37.0	0.234
The highest postoperative pain score	2.8 ± 0.4	3.1 ± 0.2	0.476
Postoperative hospital stay (days)	5.2 ± 0.8	5.8 ± 0.6	0.083
Postoperative arrhythmias	3	13	0.032
Atrial fibrillation	1	3	0.486
Tachycardia	2	10	0.039

∗ or ^#^: *p* < 0.05, the t test or Mann-Whitney *U* test was used to evaluate differences in continuous variables between the two groups. Definition of abbreviations: SD: standard deviation; IQR: interquartile range; RUL: right upper lobe; RML: right middle lobe; RLL: right lower lobe. Tachycardia standard: In the nontherapeutic state, the heart rate was >100 twice a day.

**Table 2:** Perioperative heart rate changes between experimental and control groups.

Heart rates (bpm, median (IQR))	Experimental (*n* =22)	Control (*n* =31)
Basal heart rate	77.0 (70.3-80.0)	69.5 (58.5-75.0)
Out of PACU	72.0 (68.3-77.8)	72.0 (66.3-84.0)
D 1	77.5 (72.3-83.0)	79.5 (73.0-86.5)
D 2	80.5 (72.0-88.50)	79.5 (71.0-84.8)
2 D ptd	83.5 (72.3-90.0)	78.5 (74.3-87.0)
1 D ptd	88.0 (73.8-94.0)	80.0 (75.3-87.5)

Definition of abbreviations: bpm: beats per minute; PACU: postanesthesia care unit; D 1: Day 1 after surgery; D 2: Day 2 after surgery; 2 D ptd: 2 days prior to discharge; 1 D ptd: 1 day prior to discharge.

## Data Availability

All information that does not involve patient privacy has been listed in the table at the end of the article.
